# The Effects of the Anti-aging Protein Klotho on Mucociliary Clearance

**DOI:** 10.3389/fmed.2019.00339

**Published:** 2020-01-24

**Authors:** Jaleesa Garth, Molly Easter, Elex Skylar Harris, Juliette Sailland, Lisa Kuenzi, Samuel Chung, John S. Dennis, Nathalie Baumlin, Adegboyega T. Adewale, Steven M. Rowe, Gwendalyn King, Christian Faul, Jarrod W. Barnes, Matthias Salathe, Stefanie Krick

**Affiliations:** ^1^Division of Pulmonary, Allergy and Critical Care Medicine, Department of Medicine, University of Alabama at Birmingham, Birmingham, AL, United States; ^2^Gregory Fleming James Cystic Fibrosis Research Center, The University of Alabama at Birmingham, Birmingham, AL, United States; ^3^Division of Pulmonary, Allergy, Critical Care and Sleep Medicine, Department of Medicine, University of Miami Miller School of Medicine, Miami, FL, United States; ^4^Division of Pulmonary, Critical Care, and Sleep Medicine, Department of Internal Medicine University of Kansas Medical Center, Kansas City, KS, United States; ^5^Department of Biology, Creighton University, Omaha, NE, United States; ^6^Division of Nephrology, Department of Medicine, The University of Alabama at Birmingham, Birmingham, AL, United States

**Keywords:** klotho, mucociliary clearance, TGF-β, airway surface liquid volume, calcium activated potassium channels

## Abstract

α-klotho (KL) is an anti-aging protein and has been shown to exert anti-inflammatory and anti-oxidative effects in the lung and pulmonary diseases such as chronic obstructive pulmonary disease (COPD) and cystic fibrosis. The current study investigated the direct effect of KL on the bronchial epithelium in regards to mucociliary clearance parameters. Primary human bronchial and murine tracheal epithelial cells, cultured, and differentiated at the air liquid interface (ALI), were treated with recombinant KL or infected with a lentiviral vector expressing KL. Airway surface liquid (ASL) volume, airway ion channel activities, and expression levels were analyzed. These experiments were paired with *ex vivo* analyses of mucociliary clearance in murine tracheas from klotho deficient mice and their wild type littermates. Our results showed that klotho deficiency led to impaired mucociliary clearance with a reduction in ASL volume *in vitro* and *ex vivo*. Overexpression or exogenous KL increased ASL volume, which was paralleled by increased activation of the large-conductance, Ca^2+^-activated, voltage-dependent potassium channel (BK) without effect on the cystic fibrosis transmembrane conductance regulator (CFTR). Furthermore, KL overexpression downregulated IL-8 levels and attenuated TGF-β-mediated downregulation of LRRC26, the γ subunit of BK, necessary for its function in non-excitable cells. In summary, we show that KL regulates mucociliary function by increasing ASL volume in the airways possibly due to underlying BK activation. The KL mediated BK channel activation may be a potentially important target to design therapeutic strategies in inflammatory airway diseases when ASL volume is decreased.

## Introduction

As part of the innate defense mechanism, mucociliary clearance (MCC) protects the airway epithelium by trapping inhaled pathogens or particulate matter within the mucus layer and removing it from the airways through ciliary movement (1, 2). Proper function of MCC depends on both mucus production and mucus transport, which are affected by coordinated ciliary beating, sufficient ASL volume, and mucus viscosity (3). MCC can become compromised by dysregulation of any of these key components, which renders the airway and especially the airway epithelium susceptible to infection. By failing to transport mucus effectively, airways become obstructed which leads to inflammatory changes as commonly seen in diseases such as cystic fibrosis (CF), asthma or COPD (4–9).

ASL volume is regulated by ion fluxes across the apical airway epithelial membrane, which achieves a balance of sodium (Na^+^) absorption and chloride (Cl^−^) secretion (10–12). The CFTR channel in part regulates chloride movement across the apical airway epithelial membrane to maintain airway fluid homeostasis and proper ciliary beating (13, 14). However, apical potassium (K^+^) secretion via BK channels has been increasingly recognized for its essential role in delivering an electrochemical driving force for apical chloride ion exit through CFTR and calcium-activated chloride channels (CaCC) (15). This regulated ion flux has been shown to help maintain ASL volume and MCC (16–19), and dysregulation of these channels has been documented in the pathogenesis of a multitude of inflammatory airway diseases including CF and COPD (19–21).

The klotho protein (KL) exists in several forms including the full-length membrane form and a soluble circulating form, which results from either proteolytic cleavage or alternative splicing (22, 23). KL's interaction with fibroblast growth factor (FGF) 23 is well-documented: FGF23 and KL bind to FGF receptor 1 as a co-receptor, which has been shown to regulate phosphate and calcium homeostasis in the kidney and parathyroid gland (24). In addition, KL has been characterized as an anti-aging protein, exerting anti-oxidative, anti-inflammatory, and anti-proliferative functions in the heart, lung, and kidney (25–28). We have previously shown that KL can protect the bronchial epithelium against transforming growth factor (TGF)-β-induced inflammation in CF lung disease (26). However, the role of KL on mucociliary clearance has not been evaluated. In this study, we investigated the effects of KL on ion flux across the airway epithelium and thereby ASL homeostasis.

## Methods

### Air Liquid Interface (ALI) Cell Culture

Human bronchial epithelial cells from individuals without significant lung disease (HBEC) were isolated and cultured using the ALI model as described previously (19, 29). Institutional review board-approved consent for research was obtained by the Life Alliance Organ Recovery Agency of the University of Miami or the Life Center Northwest and the University of Alabama at Birmingham.

Murine tracheal epithelial cells (MTEC) from wild type mice and mice, homozygous for the klotho gene disruption (30), were isolated, cultured, and differentiated for 2–3 weeks according to an adapted protocol of You et al., as previously described (26, 31, 32).

### μOCT Analyses

The μOCT technique and analysis have been described previously (33–35). Briefly, CBF, MCT, and ASL height were directly evaluated via cross-sectional images of the airway epithelium using high acquisition speed and high resolution. Quantitative analysis of the images was achieved by use of ImageJ (36).

### Airway Surface Liquid (ASL) Volume *in vitro*

ASL volumes from HBECs and MTECs were quantified by meniscus scanning and data were analyzed using the software generously provided by Dr. Myerburg (University of Pittsburgh) (37).

### Electrophysiology

Differentiated HBECs on Snapwell filters were mounted in Ussing chambers (Easymount chamber; Physiologic Instruments) connected to a VCC MC6 voltage clamp unit (Physiologic Instruments, San Diego, CA, USA) as previously described (37). For BK activity, basolateral membranes were permeabilized for 30 min with 20 μM amphotericin B, 10 μM nigericin, and 10 μM valinomycin (whole cell short circuit current recordings do not distinguish K^+^ efflux but measure net current, a combination of K^+^ and Cl^−^ efflux) (16). For assessment of BK currents, cells were exposed to a K^+^ gradient in the presence of apically applied 10 μM amiloride (Sigma-Aldrich #A7410, St. Louis, MO, USA) and 10 μM ATP (Sigma-Aldrich #A1852). CFTR activity was assessed in non-permeabilized cells using apical 5 mM Cl^−^ in the presence of apically applied 10 μM amiloride and 10 μM forskolin (Sigma-Aldrich #F3917) followed by 10 μM CFTR_inh_172 (Sigma-Aldrich #C2992) as described previously (14, 37).

### ELISA

An ultrasensitive IL-8 enzyme-linked immunosorbent assay (ELISA) from Invitrogen (Thermo Fisher, Waltham, MA, USA) was used as described previously (26).

### Murine *Kl* Overexpression in NHBEC Using a Lentiviral Expression System

A full length murine α-klotho, which was kindly provided by Dr. Kuro-o (38), was cloned into a p38 plasmid containing a puromycin resistance cassette. Lentiviral infection of normal HBEC and puromycin selection was done before differentiation as previously described (26, 32, 39).

### Intracellular Calcium Imaging Using GCaMP6s Sensor

Imaging was performed as previously described (40). A pEF1-Puromycin-expressing GCaMP6s construct was designed using pGP-CMV-GCaMP6s (Addgene plasmid #40753) gifted by Dr. Douglas Kim (41). NHBEC cultures were infected in an undifferentiated state with packaged lentiviruses to deliver pEF1-GCaMP6s. Cultures were allowed to fully-differentiate at the air-liquid interface (>4 weeks) under constant puromycin selection (1 μg/ml). GCaMP6s-expressing cultures were perfused at room temperature with HEPES-buffered HBSS, pH 7.3 at 250 μL min^−1^ (42). GSK1016790A (Tocris), HC-067047 (Tocris), α-Klotho (Peprotech), and DMSO vehicle control (0.1%; Sigma-Aldrich) were dissolved in HEPES-buffered HBSS and also perfused at 250 μL min^−1^. GCaMP6s emissions were recorded every 3 s using MetaFluor (Molecular Devices). Data were analyzed as relative calcium levels (F_x_/F_0_) using IGOR software (WaveMetrics).

### Statistics

Experimental data were analyzed with Prism8 (GraphPad Software, Inc., La Jolla, CA) as previously described (19) using Student's *t* test and analysis of variance or Kruskal Wallis with appropriate post tests for at least three independent experiments. Significance was accepted at *p* < 0.05.

## Results

### ASL Height and Volume Is Significantly Decreased in Tracheas From *kl^−/−^* Deficient Mice

**Figure 1:**
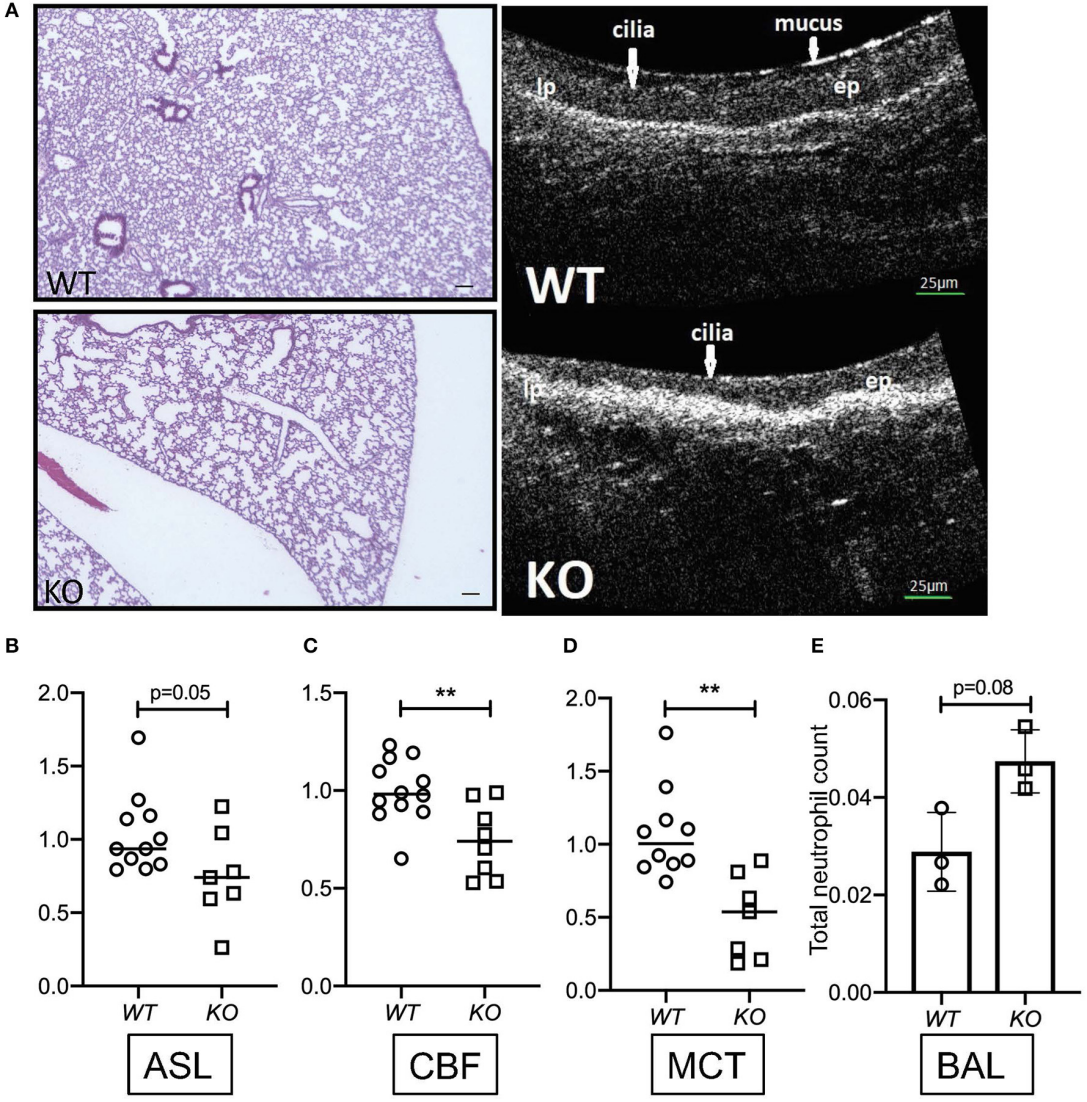
**(A)** Representative images showing hematoxylin staining of whole lung sections from *kl*^−/−^ (KO) and *kl*^+/+^ (WT) mice (4X magnification, scale bar = 100 μm) and representative images of μOCT recordings from *kl*^−/−^ (KO) and *kl*^+/+^ (WT) tracheas. **(B)** Comparison of fold changes in airway surface liquid (ASL) depth, **(C)** ciliary beat frequency (CBF), and **(D)** mucociliary transport *ex vivo* from excised tracheas of *kl*^−/−^ (KO) and *kl*^+/+^ (WT) mice using μOCT. **(E)** Bronchoalveolar lavage fluid (BALF) analysis showing a trend increase of total neutrophil cell count in three representative WT and KO mice. Statistics: Student's *t*-test showing mean ± S.D. with ^**^p < 0.01, as indicated in appropriate graphs (*n* = 7–11- animals per group).

To determine the relevance of KL on parameters of mucociliary function, we harvested tracheas from *kl*^−/−^ mice and their wild type littermates, analyzing them using μOCT as previously described (3). Consistent with previous findings, we observed dilated airway spaces, consistent with emphysema, when compared to wild type littermates (Figure 1A). Interestingly, there was a significant decrease in ASL depth in the *kl*^−/−^ mice (Figure 1A right panel showing μOCT images and Figure 1B), and decreased ciliary beat frequency (CBF) and mucociliary transport (MCT) (Figures 1C,D). Previously, we and others have shown that *kl*^−/−^ mice have a significant increase in total cell count and macrophage/monocytes in bronchoalveolar lavage (BAL) fluid indicating airway inflammation (43). Consistent with these previous findings, we show here also increased neutrophils in BAL fluid from *kl*^−/−^ lungs when compared to wild type lungs (Figure 1E). In summary, klotho deficient mice show emphysema, lung inflammation and a decrease in ASL depth, CBF resulting in impaired MCT.

### *In vitro* Effects of Klotho on ASL Volume Regulation

**Figure 2:**
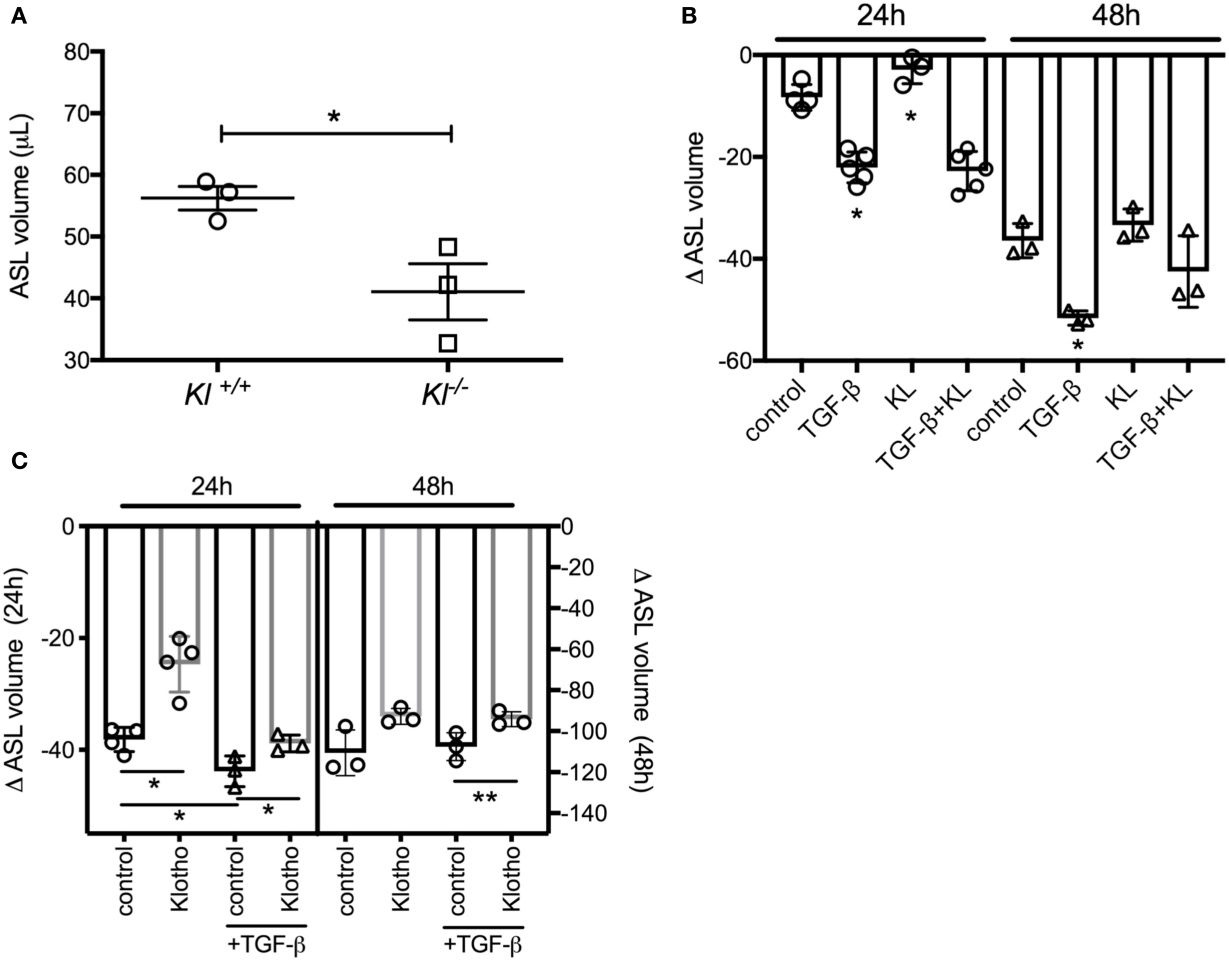
**(A)** Dot plot showing a significant decrease of ASL volume in murine tracheal epithelial cells (MTECs), isolated from *kl*^−/−^ mice and their wild type littermates and using meniscus scanning. **(B)** Bar graphs indicating ASL volume change in human bronchial epithelial cells (HBEC), differentiated at the ALI interface and treated with TGF-β (2.5 ng/ml) ± recombinant KL (100 ng/ml) for 24 and 48 h. **(C)** ASL volume change in HBEC, infected with either control or klotho and differentiated at the ALI interface and effect of TGF-β (2.5 ng/ml) for 24 and 48 h. (*n* = 3 independent experiments showing mean ± S.E. with ^*^P < 0.05 and ^**^P < 0.01).

To validate our *ex vivo* data, we isolated primary murine tracheal epithelial cells (MTECs) and differentiated them at the ALI for 3–4 weeks until cilia and mucus were present as shown previously (43). MTECs, isolated from *kl*^−/−^ mice also showed a significant decrease in their baseline ASL volume (Figure 2A). When primary human bronchial epithelial ALI cultures (HBEC) from control lungs were stimulated with TGF-β, there was a significant decrease in ASL volume, consistent with the known deleterious effects of TGF-β signaling on ion transport in non-CF epithelia (44). Supplementation of these cultures with human recombinant klotho protein mildly increased ASL volume after 24 h but did not attenuate the TGF-β response within 24 or 48 h (Figure 2B). Since we experienced significant loss of activity of the recombinant klotho protein after short storage time or a freeze thaw cycle, we developed a lentiviral overexpression system of murine full length klotho in our ALI cultures (26). Assessment of these differentiated klotho overexpressing ALI cultures showed a significant increase in ASL volume after 24 h, when compared to control-infected ALI cultures. Additionally, the TGF-β-mediated reduction in ASL volume was also attenuated in the klotho overexpressing cultures at 48 h (Figure 2C).

### *In vitro* Effects of Klotho on HBEC Ion Channel Activation and Expression

**Figure 3:**
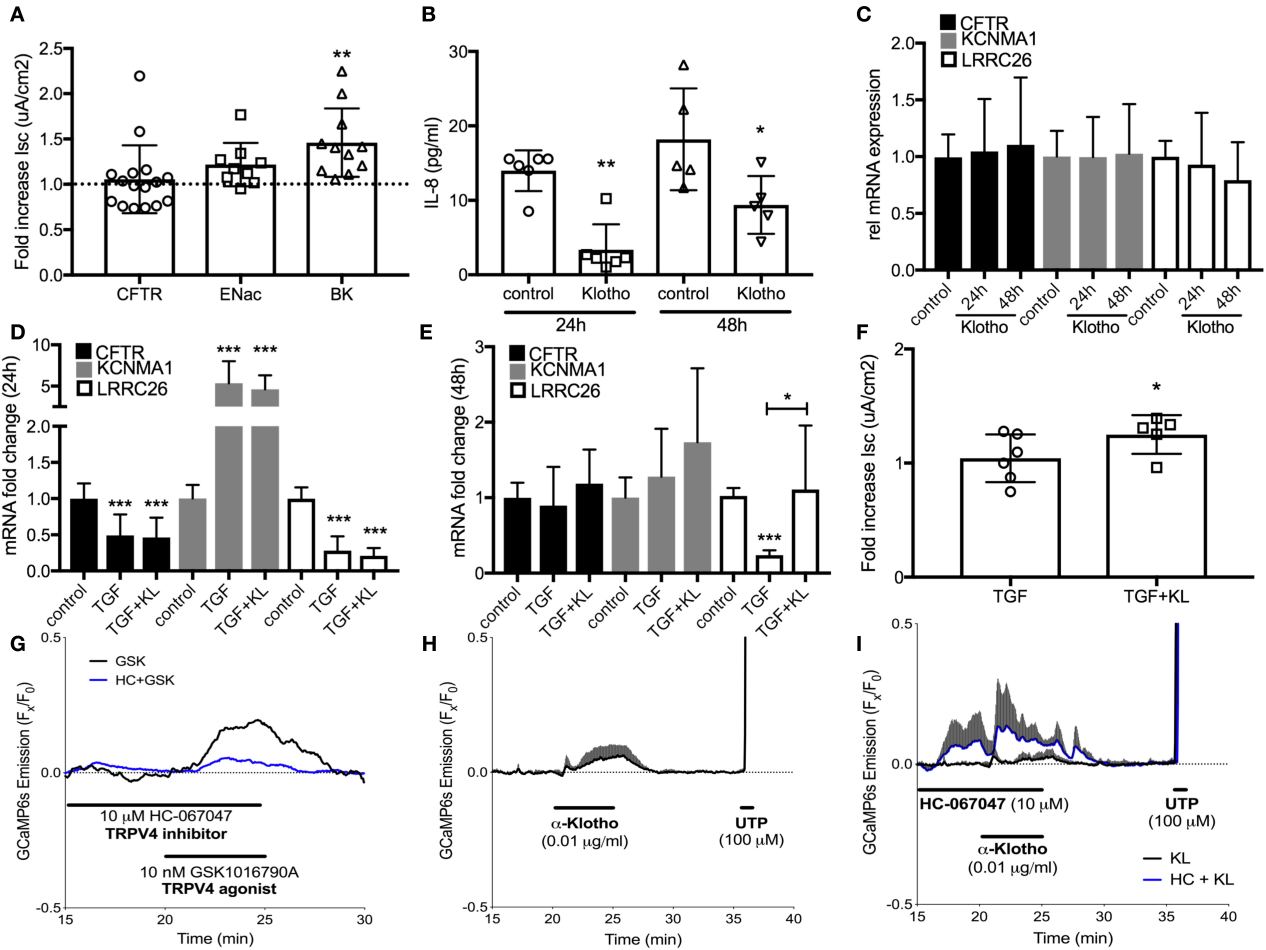
**(A)** Effect of treatment with recombinant KL on the activation of CFTR, ENaC, and BK channels, assessed in human ALI cultures using Ussing chambers. **(B)** Effect of klotho overexpression on basolateral IL-8 secretion in HBECs. **(C)** Bar graphs indicating changes in relative mRNA expression of CFTR and BK channels subunits after treatment with KL, **(D)** TGF-β (2.5 ng/mL) after 24 h and **(E)** 48 h. **(F)** Effect of TGF-β ± KL on BK channel conductance in HBEC. **(G)** 10 nM GSK1016790A, a TRPV4-selective agonist, transiently increases intracellular calcium in GCaMP6s expressing HBECs. This response is partially blocked in the presence of TRPV4 antagonist HC-067047 (10 μM, *n* = 1). **(H)** Acute KL exposure (0.01 μg/ml, 5 min) transiently increases intracellular calcium (*n* = 3). **(I)** Pre-exposure to HC-067047 amplified KL-mediated calcium effects. 100 μM UTP served as a positive control for calcium influx (*n* = 3 independent experiments from 3 to 5 different lungs showing mean ± S.E. with ^*^P < 0.05, ^**^P < 0.01, and ^***^P < 0.005).

**Figure 4:**
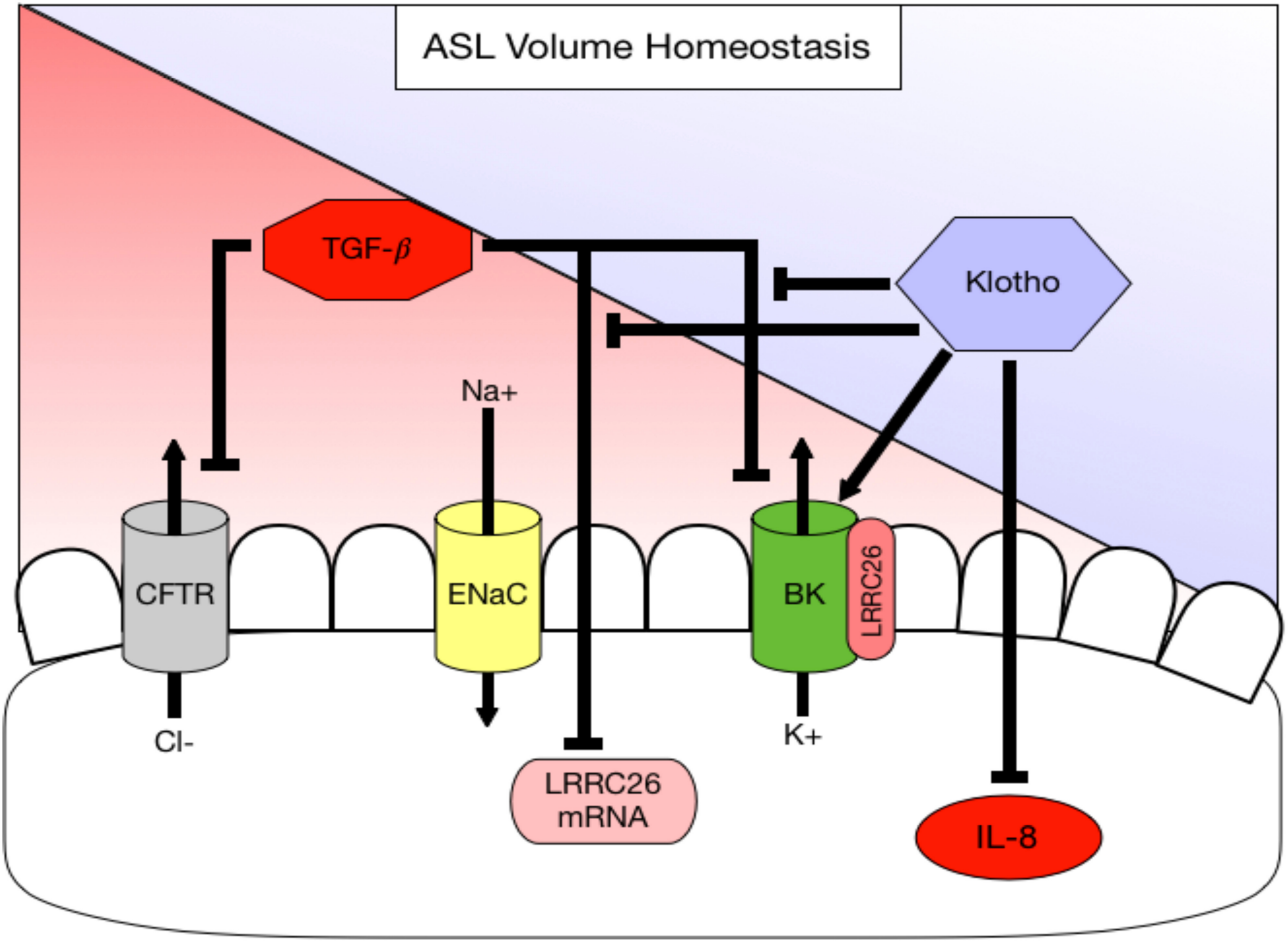
Diagram summarizing working hypothesis. Klotho exerts an activating effect on the apical BK channel through a direct action but also indirectly by attenuating (1) a TGF-β-mediated decrease in LRRC26, the regulatory subunit of the BK channel and (2) a TGF-β-mediated inhibition of BK. In addition, klotho decreases basolateral IL-8 secretion, which is pro-inflammatory thereby contributing to ASL volume dysregulation.

To assess underlying mechanisms how klotho regulates ASL volume, we used Ussing chamber measurements to assess CFTR, BK, and ENaC activities; all channels expressed in ALI cultures and contributing to ASL volume regulation (10, 16). When ALI cultures were stimulated with recombinant KL or infected with KL lentiviral particles, only BK channel activity improved significantly at 24 h (Figure 3A). Lentiviral overexpression of KL also led to a persistent decrease of IL-8 secretion in these cultures (Figure 3B). KL itself neither changed mRNA expression of CFTR, KCNMA1, and LRRC26 (two BK channel subunits) (Figure 3C), nor affected TGF-β mediated changes after 24 h (Figure 3D), but there was attenuation of TGF-β-induced reduction in LRRC26 mRNA levels after 48 h (Figure 3E). This attenuation was also noted in the analysis of BK channel activity (Figure 3F). To further elucidate potential underlying mechanism for the described klotho effects, we could show that recombinant klotho transiently increased intracellular calcium in NHBEC cultures, comparable to a TRPV4 channel selective agonist (GSK1016790A) (Figures 3G,H). Pre-exposure to the TRPV4 inhibitor amplified the klotho effect on calcium further (Figure 3I). In summary, klotho attenuated IL-8 secretion in ALI cultures and may activate and partially restore the BK channel following TGF-β treatment with intracellular calcium increase as a potential underlying mechanism, providing protection from a pro-inflammatory environment (Figure 4).

## Discussion

α-Klotho is an anti-aging protein, originally known from its expression in the kidney (30). Mice deficient in klotho develop an aging phenotype including emphysematous lung changes and airway inflammation. We have previously shown that klotho exerts an anti-inflammatory action in cystic fibrosis airway disease by counteracting TGF-β signaling (26), but it is not clear how klotho affects the mucociliary transport apparatus. This study examines for the first time the role of klotho on mucociliary clearance *ex vivo* and *in vitro* and attempts to identify potential underlying mechanisms. Our results show that CBF was not affected in the klotho deficient mouse model, but that ASL volume, CBF and MCT are significantly reduced *ex vivo* and *in vitro*, which is paralleled by neutrophilic airway inflammation. In addition, we show that overexpression of klotho increased ASL volume. In order to identify a potential underlying mechanism, we showed that IL-8 levels were attenuated in klotho-overexpressing ALI-cultures. This is important, since IL-8 has been shown to negatively regulate ASL volume (10, 17, 45). Furthermore, klotho itself can increase intracellular calcium and affects BK channel activity, an apical potassium channel that has been shown to be involved in ASL homeostasis together with CFTR (16, 19). One potential mechanism could be through restoration of its regulatory subunit LRRC26 (Figure 4).

Klotho is expressed in the lung, but it is downregulated in airway diseases such as COPD (27, 43, 46). Interestingly, other reports have described an absence of klotho expression in the lung and suggested that it is synthesized in the kidney, cleaved at the transmembrane domain, released into the circulation and taken up by the lung (47, 48). Thus, more studies are needed to determine the exact organ genesis of klotho. However, the susceptibility to degradation and lack of sufficient tools to detect klotho make it difficult to study presently. Therefore, it was challenging for our studies to demonstrate overexpressed murine klotho, which made us therefore use both overexpression and exogenous stimulation for our studies. Using these approaches, there is sufficient evidence to prove that klotho clearly exerts effects on the lung and airways. Currently, klotho signaling is mainly linked to FGF23 signaling, where klotho functions as a co-receptor mediating phosphorylation of ERK (49). Recent studies suggest that there is not only klotho independent FGF23 signaling (43, 50) but also FGF23 independent klotho signaling (51, 52). We are aware that klotho deficient mice have elevated FGF23 levels, which could be responsible for mucociliary dysfunction, but we conducted all our *in vitro* assays in ALI cultures that do not express FGF23. Therefore, the klotho-mediated effects on mucociliary clearance should be independent of FGF23. We propose that the effect of klotho is two-fold: (1) klotho can directly activate the BK channel (Figure 3A) by an unknown mechanism, possibly through an increase of intracellular calcium (40); and (2) klotho can rescue the TGF-β-mediated downregulation of LRRC26 and restore BK function (Figures 2, 3E). More mechanistic studies are needed to identify the exact signaling pathway. Importantly, these discoveries open new avenues of research to find anti-inflammatory and anti-aging therapies for restoring klotho levels or increasing klotho signaling in the airway through the improvement of ASL volume and mucociliary clearance. These therapies would potentially benefit a variety of diseases that feature acute or chronic airway inflammation.

## Data Availability Statement

The datasets generated for this study are available on request to the corresponding author.

## Ethics Statement

All experiments were approved by the University of Miami and University of Alabama at Birmingham (UAB) Institutional Animal Care and Use Committee (IACUC), and mice were housed in a UM or UAB facility approved by the Association for Assessment and Accreditation of Laboratory Animal Care (AAALAC). Written informed consent was obtained from the participants of this study. Standard biosecurity and institutional safety procedures have been adhered to.

## Author Contributions

SK and MS contributed to the concept and/or design of the study. SK, JG, ME, ES, JS, LK, GK, SC, JD, NB, and AA contributed to the acquisition of the data. SK, JG, CF, SR, JB, and MS contributed to the analysis and interpretation. JG and SK drafted the manuscript. All authors critically revised it for intellectual content and approved the final version prior to submission.

### Conflict of Interest

The authors declare that the research was conducted in the absence of any commercial or financial relationships that could be construed as a potential conflict of interest.
